# Retracted: Chronic Recurrent Multifocal Osteomyelitis with Concomitant Features of Juvenile Idiopathic Arthritis

**DOI:** 10.1155/2014/341304

**Published:** 2014-08-28

**Authors:** Case Reports in Rheumatology

The paper titled “Chronic recurrent multifocal osteomyelitis with concomitant features of juvenile idiopathic arthritis” [1], published in Case Reports in Rheumatology, has been retracted as it was submitted for publication without receiving the patient's consent to be included in the study.
